# Cardiac Magnetic Resonance in Association With Coronary CT Angiography in the Assessment of Right Coronary Artery Aneurysm With Atrial Fistula Causing Myocardial Ischemia

**DOI:** 10.7759/cureus.77218

**Published:** 2025-01-10

**Authors:** Mohammed Mousa, Islam Z Mahmoud, Tarek H Elkammash, Azza A Gad, Radwa A Noureldin

**Affiliations:** 1 Department of Diagnostic and Interventional Radiology, Suez Canal University, Ismailia, EGY; 2 Department of Cardiovascular Medicine, Suez Canal University, Ismailia, EGY

**Keywords:** cardiac magnetic resonance (cmr), coronary ct angiography, coronary fistula, myocardial ischemia, right coronary artery aneurysm

## Abstract

Coronary artery aneurysms are uncommon. Atherosclerosis is the primary etiology, and the right coronary artery (RCA) is the most frequently involved. We present a case of a 65-year-old male patient presenting with ischemic symptoms and a giant right coronary artery aneurysm with a fistula to the right atrium. The diagnosis was established using non-invasive imaging modalities, including cardiac magnetic resonance (CMR) and coronary computed tomography angiography (CCTA). This case underscores the critical role of a combined imaging approach in effectively managing such rare and complex coronary artery conditions. The collaboration of CMR, providing data on myocardial viability and thrombus, with CCTA, offering precise anatomical details, enables clinicians to gain a comprehensive understanding of the aneurysm and its complications. This integrated approach guides optimal treatment strategies and ultimately improves patient outcomes.

## Introduction

Giant coronary artery aneurysms, defined as those exceeding 5 cm in diameter, represent a rare subset of coronary artery aneurysms with an estimated incidence of 0.02%-0.04% [1]. The right coronary artery (RCA) is the most frequent site of involvement [2]. The precise mechanisms underlying the development of both coronary artery aneurysms and coronary artery fistulas remain incompletely understood. Atherosclerosis is the predominant cause of coronary artery aneurysms in adults. However, coronary artery aneurysms can also arise in the context of congenital heart defects, Kawasaki disease, vasculitides such as Takayasu arteritis, and connective tissue diseases [3]. While most individuals remain asymptomatic, these lesions can manifest with potentially life-threatening complications, including myocardial infarction, fistula formation, and congestive heart failure [4]. This case report describes a patient with a giant coronary artery aneurysm with a fistula to the right atrium who presented with a history of exertional dyspnea and chest tightness. This study will emphasize the valuable contributions of cardiac magnetic resonance (CMR) imaging, which was requested for tissue characterization and provided data on myocardial viability and aneurysm thrombosis, and coronary computed tomography angiography (CCTA), which offered precise anatomical details, to the diagnostic process.

## Case presentation

A 65-year-old male patient with a history of chronic obstructive pulmonary disease, uncontrolled hypertension, and type 2 diabetes mellitus presented to our department with a two-month history of exertional dyspnea and chest tightness. Transthoracic echocardiography was limited by suboptimal acoustic window secondary to severe emphysema. Despite these limitations, the echocardiogram revealed a large mass lesion adjacent to the right cardiac border. This mass appeared to be compressing the right atrium and measured approximately 6×5.5 cm. Due to a suboptimal acoustic window, transthoracic echocardiography was unable to adequately characterize the reported mass lesion or determine its origin. Therefore, further cardiac magnetic resonance (CMR) was recommended for better delineation and tissue characterization.

CMR findings

**Figure 1:**
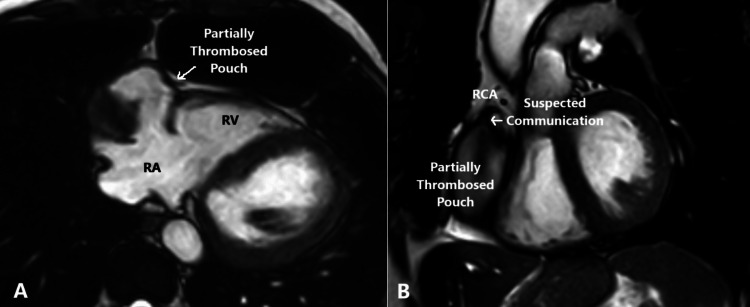
A: Large pouch connected to the right atrium with a hypointense lesion seen at its posterior segment (arrow). B: Suspected communication between the RCA and the previously described pouch (arrow). RCA: right coronary artery, RA: right atrium, RV: right ventricle

A large outpouching is noted related to the right atrium lateral wall (Figure 1A) measuring 51×43×64 mm in maximum axial and cranio-caudal dimensions (anterior-posterior × transverse × cranio-caudal), connected to the right atrium through a wide neck measuring 3×2.1 cm. Suspected communication is seen between the proximal part of the RCA and the outpouching (Figure 1B). This outpouching is seen akinetic through the cardiac cycle. This akinesis has potential implications for perfusion within the outpouching and may increase the risk of thrombus formation.

**Figure 2:**
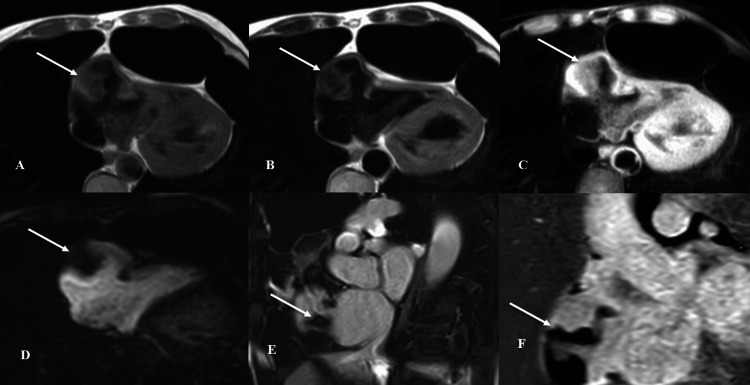
Multi-sequence CMR imaging characterizes the aforementioned lesion. A: T1WIs reveal isointensity (arrow). B: T2WIs depict hypointensity (arrow). C: The absence of signal dropout on FS WIs (arrow). D: No contrast uptake in perfusion images (arrow). E and F: EGE and PSIR images show a dark signal suggesting thrombosis within the lesion (arrow). CMR: cardiac magnetic resonance, TSE: turbo spin echo, EGE: early gadolinium enhancement, PSIR: phase-sensitive inversion recovery, T1WI: T1-weighted image, T2WI: T2-weighted image, FS WI: fat-suppressed image

A hypointense lesion is seen within its posterior segment measuring 33×25×33 mm (anterior-posterior × transverse × cranio-caudal) demonstrating an isointense signal in T1WIs, a hypointense signal in T2WIs, no signal drop in fat suppression images, no contrast uptake in perfusion images with a dark signal with no significant enhancement in late gadolinium inversion recovery, and phase-sensitive inversion recovery images suggesting thrombosis (Figure 2). The presence of a thrombus within the outpouching may be clinically significant, potentially increasing the risk of embolization and related complications.

**Figure 3:**
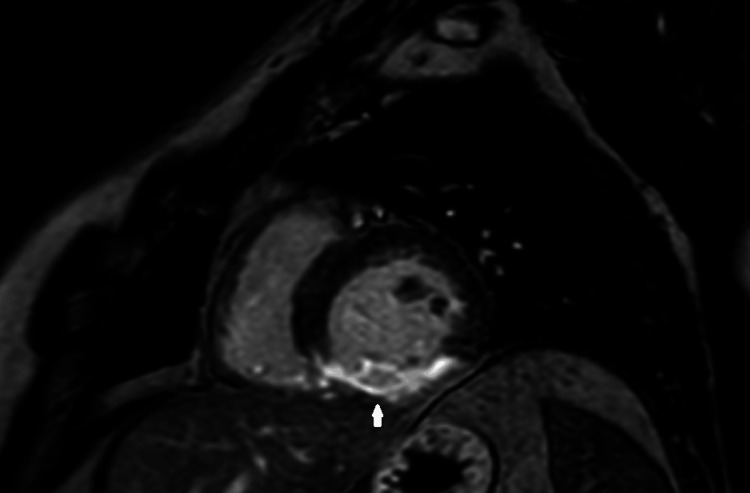
Late gadolinium enhancement inversion recovery image shows transmural scarring (arrow) along inferior segments (RCA territory). RCA: right coronary artery

Late gadolinium inversion recovery images over the left ventricle show transmural infarction along RCA territory myocardial segments at inferior segments (Figure 3).

Coronary CT angiography findings

**Figure 4:**
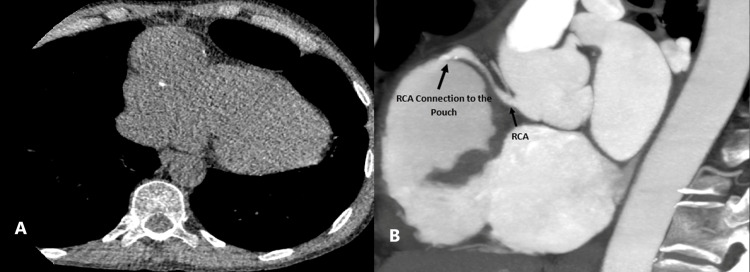
A: Faint wall calcifications were observed within the aneurysm on non-contrast images. B: Communication with the RCA was demonstrated on MIP sagittal images (arrow). RCA: right coronary artery, MIP: maximum intensity projection

Coronary CT angiography (CCTA) revealed an outpouching with faint wall calcifications evident on non-contrast images. The presence of faint wall calcifications may indicate a chronic nature of the outpouching or suggest an atherosclerotic etiology for the condition. This outpouching is seen connected to both the proximal and distal thirds of the right coronary artery as shown in Figure 4. This connection may have implications for coronary blood flow dynamics, potentially leading to altered hemodynamics within the RCA and increasing the risk of ischemia in the myocardial segments supplied by the RCA.

## Discussion

Coronary artery aneurysms are detected in approximately 0.9%-4.9% of patients undergoing coronary angiography. The right coronary artery is the most commonly affected vessel [2].

Atherosclerosis constitutes the primary etiological factor for the development of coronary artery aneurysms. This pathological process induces mechanical stress on the vessel wall [5], triggering an inflammatory response characterized by the degradation of extracellular matrix proteins through the activation of proteolytic enzymes [6]. Within the aneurysm, an imbalance arises, with elevated levels of matrix metalloproteinases (MMPs) and decreased levels of tissue inhibitors. This heightened MMP activity results in the degradation of the arterial media, leading to a thinning of the vessel wall and increased wall stress, ultimately contributing to arterial dilatation [6].

Our patient exhibited no prior history of chest trauma, Kawasaki disease, or connective tissue disorders. Furthermore, CCTA demonstrated the presence of wall calcific plaques, suggesting that the underlying etiology of his aneurysm was atherosclerotic disease, the most common pathologic basis for coronary artery aneurysms.

Single coronary artery fistulas demonstrate a higher prevalence (74%-90%) compared to multiple fistulas (10.7%-16%) [7]. A majority of these fistulas originate from the right coronary artery (RCA), with an incidence reported between 50% and 60%. Regarding drainage sites, a significant proportion (15%-43%) connect to the pulmonary artery, followed by the right atrium (19%-26%) and right ventricle (14%-40%). Drainage into the left ventricle is less frequent, occurring in only 2%-19% of cases, as documented across various studies [7,8].

Coronary angiography remains the gold standard for definitive evaluation. CCTA offers significant value in the assessment of patients with giant coronary artery aneurysms. Its primary advantage lies in its exceptional three-dimensional visualization capabilities, which can mitigate the risk of underestimating aneurysm size in cases with partial thrombosis. Furthermore, CCTA provides a detailed description of the involved coronary artery, including its side branches and distal extensions, as well as a comprehensive overview of surrounding anatomical structures [9,10].

CMR enables the assessment of myocardial viability. In cases where the coronary artery aneurysms present with partial thrombosis, CMR can aid in differentiating the aneurysm from a cardiac tumor [11,12].

This case highlights the complementary roles of cardiac magnetic resonance (CMR) imaging and coronary computed tomography angiography (CCTA) in the diagnosis and management of this complex patient.

CMR provided crucial insights by assessing myocardial viability. CMR helped evaluate the impact of the aneurysm and potential ischemia on myocardial function, aiding in risk stratification. It also helped in detecting aneurysm thrombosis.

CCTA played a crucial role in precisely delineating the aneurysm's anatomy. It accurately visualized the fistula between the right coronary artery and the right atrium and assessed the extent of coronary artery involvement. CCTA allowed for a comprehensive evaluation of the coronary arteries, including the identification of associated atherosclerotic plaque and calcifications, which provided valuable insights into the patient's overall cardiovascular risk.

This multidisciplinary approach, involving cardiologists, radiologists, and surgeons, facilitated a comprehensive understanding of the patient's condition and enabled the selection of the most appropriate management strategy.

Given the patient's multiple comorbidities, along with the findings provided by CMR and CCTA including non-viable myocardium within the right coronary artery (RCA) perfusion territory, a conservative management strategy with close follow-up was deemed most appropriate.

## Conclusions

This case underscores the critical role of advanced cardiac imaging, specifically cardiac magnetic resonance (CMR) imaging and coronary computed tomography angiography (CCTA), in the diagnosis and management of a rare case of a giant right coronary artery aneurysm with an atrial fistula. CCTA provided precise anatomical details of the aneurysm, while CMR offered valuable insights into myocardial viability and helped identify areas of scarring and thrombosis. Given the patient's multiple comorbidities and the presence of non-viable myocardium within the right coronary artery perfusion territory as demonstrated by CMR and CCTA, a conservative approach with regular follow-up was determined to be the best course of action for this patient.

This case emphasizes the importance of a multidisciplinary approach, integrating advanced imaging findings with clinical expertise, in the successful management of complex cardiovascular pathologies.
